# Urban Vitality Measurement and Influence Mechanism Detection in China

**DOI:** 10.3390/ijerph20010046

**Published:** 2022-12-21

**Authors:** Jinghu Pan, Xiuwei Zhu, Xin Zhang

**Affiliations:** 1 College of Geography and Environmental Science, Northwest Normal University, Lanzhou 730070, China; 2 Lanzhou Regional Climate Center, Lanzhou 730020, China

**Keywords:** urban vitality, multi-source spatial data, spatial pattern, spatial scan, multi-scale geographically weighted regression

## Abstract

Urban vitality is the life force of a city. In this paper, starting from three subsystems of population, economy, and function, the comprehensive index system for measuring urban vitality was constructed respectively from three scales: grid, prefecture-level administrative region, and urban agglomeration. GIS spatial analysis methods were used to measure the urban vitality index and analyze the spatial distribution pattern. Then, the MGWR was used to reveal the main factors affecting the difference in urban vitality and analyze the influence mechanism of urban vitality. Accordingly, countermeasures and suggestions for creating vibrancy were put forward. The result shows the following: At the grid scale, urban vitality presents a spatial distribution pattern of “large dispersion, small agglomeration”, which has significant differentiation characteristics of city scale and hierarchy. At the administrative region scale, the overall vitality of cities at the prefecture level and above in China is not high, and the spatial differences are large. The spatial scan identified 28 vigorous cities with high potential, belonging to 6 vigorous clusters. On the scale of urban agglomeration, according to the degree of vitality, there are three gradients. The spatial difference in urban vitality was affected by the internal characteristics and external environment.

## 1. Introduction

Vitality is synonymous with the beauty of a city and the internal driving force of urban development. The worldwide urbanization process continues to advance, constantly updating the global economic and social structure. China’s urbanization is developing rapidly, but it seems to have entered a watershed [1]; the population and resources are concentrated in the megalopolis and urban agglomeration, which have vitality. With central cities and urban agglomeration as the main body, the dynamic polarization of development is becoming increasingly serious, and cities are experiencing both “decline” and “revitalization”. According to the seventh National Census of China, from 2011 to 2020, 184 of China’s 339 cities at prefecture level or above maintained population growth, most of them were vibrant national central cities, populations in Shenzhen, Guangzhou, Chengdu, and Xi’an had increased 7.13, 5.97, 5.81, and 4.48 million in 10 years, and Zhuhai, Sanya, Zhongshan, Jiayuguan, Foshan, Jinhua, Huizhou, and other prefecture-level cities had also entered the ranks of the cities with the highest increase in “attracting people” in China. Meanwhile, the population of 154 cities were shrinking, such as Suihua and Qiqihar in Heilongjiang Province that had both shrunk by more than 1 million. All these indicate that the population is further concentrated in vibrant cities and urban agglomeration. On the other hand, the pure pursuit of the increase in the number of cities and the expansion of the city scale has led to the coexistence of “precious cities” and “ghost cities”, and the emergence of “empty cities” and “abandoned cities” in many places in China, which has weakened the vitality of cities and hindered the healthy development of cities. “Precious cities” refers to cities with good comprehensive strength development but with high housing prices, “ghost cities” refers to cities with high vacancy rates due to blind expansion, and “empty cities” and “abandoned cities” refer to cities with few residents or even abandoned ones. Therefore, how to promote high-quality urban development and build a vibrant city has become the key problem that every city needs to think about [2].

China’s urbanization process is in the transition stage from the extensive development of scale expansion to the connotative development of quality improvement. In order to coordinate the speed and quality of development, cities must aim at improving the quality and vitality of cities. As an important symbol representing the level and potential of urban development, urban vitality plays an important role in the process of new urbanization. How to build a vibrant city has become a hot topic in research, and the premise and basis of the research are to objectively and comprehensively evaluate the vitality of the city. The National “14th Five-Year Plan” of China aims to comprehensively improve the quality of the city, and for the first time was put forward to “implement urban renewal action”. It will obtain a clear picture of urban development, explore the spatial direction and mode of urban renewal, and identify weaknesses in urban development through scientific evaluation of the spatial pattern of urban vitality; it is of great significance in the transformation period of urban development in China.

In 1961, Jacobs [3] put forward the concept of “urban vitality” for the first time, pointing out that urban vitality comes from the interaction between people and people, and between people and living space. Since then, scholars have carried out a lot of studies on the concept and theory of urban vitality, the evaluation of urban vitality, identification of a spatial pattern of urban vitality, and influence mechanism of urban vitality. In the conception studies of urban vitality, Ford [4] draws on the concept of urban vitality to explain new urban planning. Gehl [5] pointed out the root of urban vitality is social activities in cities. Montgomery [6] believes that urban vitality refers to the uninterrupted flow of people, convenient facilities, rich and diverse cultural activities and celebrations, as well as a sense of vitality. Ahmad et al. [7] believe that the economic, social, and cultural aspects of environmental vitality have a great impact on urban vitality. The city itself does not create vitality—its vitality comes from the interaction between people in the city, people, and the surrounding environment. The research on urban vitality mostly depends on people’s behavior and activities and the relationship between people and the environment. Ye and Nes [8] used GIS to quantitatively evaluate the urban spatial quality. In urban planning, Kirsch et al. [9] analyzed the suitability of urban garden sites in Houston, Texas, with geospatial methods and multiple measures. Pakz et al. [10] emphasized the multi-dimension of urban vitality, and measured the urban vitality of the reconstruction and new areas. The research scale can be divided into macro and micro.

Wang et al. [11] adopted an AHP-fuzzy comprehensive evaluation method to build the evaluation model of public space vitality of urban resettlement community. Keerthana and Bindu [12] made a systematic evaluation of the decline of urban vitality from the aspects of economy, society, and ecology. Liu et al. [13] comprehensively evaluated the vitality of 15 counties and cities in Jiangsu Province of China using two methods: the comprehensive entropy method and the fuzzy matte-element model. At the medium and micro level, it is mainly divided into urban public space [14], and urban specific-type space from the perspective of urban internal space for evaluation [15]. In the influence mechanism of urban vitality, Jacobs believes that the increase in function, scale, cultural characteristics, and population can promote the vitality of urban space [3]. Wilmoth [16] proposed that the necessary conditions for forming dynamic space are good urban form, diverse activities, and multi-functional streets. Wu et al. [17] proposed a quantifiable and reproducible framework for heritage adaptation by combining urban form and urban vitality measures from large geospatial data. Li et al. [18] found in their study that population density, community years, open space rate, sidewalk proportion, number of lighted roads, shopping and leisure density, degree of integration, and traffic proximity are positive factors inducing urban vitality, while road density, park proximity, and green space proximity have opposite effects.

Although existing studies on urban vitality have wide coverage and rich content, they still have the following deficiencies: Data source is single—mostly using statistical data, questionnaire surveys, and other single data sources—and lack of support of spatial big data, which makes it difficult to reflect the spatial difference of urban vitality. The measurement perspective is more onefold, and vitality measurement mostly adopts population and related indicators. There are few studies on a comprehensive evaluation of urban vitality from multi-dimensions, and few related studies from the perspective of geographic space. The research scale is onefold; most of which take a certain city or block as the research area, and there is a lack of multi-scale research at the national level. Urban vitality research is facing the transformation of the research paradigm and the innovation of data and methods. The rapid development and popularity of wireless communication, mobile positioning, and Internet technologies make it possible to obtain human behavior and activity patterns based on massive spatio-temporal trajectories at individual granularity [19,20]. Mobile phone signaling data [21], GPS trajectory [22], social media data [23], etc., have been gradually applied by scholars in the study of urban vitality measurement. From the perspective of space, this paper evaluates urban vitality in China from multivariate data sources, multivariate scales, and multivariate perspectives, and explores the influencing mechanism so as to provide scientific reference for enriching theoretical research on urban vitality, urban planning practice, urban quality improvement, and urban vitality shaping.

The findings of this study could provide valuable implications for the relevant research in the field of urban planning and urban development in China, as well as for the formulation of relevant policies of government agencies, thereby contributing to global research in the urban vitality measurement and other related fields of in-depth research.

## 2. Study Area, Data, and Methods

### 2.1. Study Area

China implements the system of Municipality Governing Counties, and prefecture-level cities are all economic and cultural centers of their regions. Exploring the spatial differentiation pattern of urban vitality at the overall level of prefecture-level cities has an important reference significance for formulating appropriate urban development policies. The research subjects of this paper are 367 cities at the prefecture level and above, including 4 municipalities directly under the Central Government, 293 prefecture-level cities, 30 autonomous prefectures, 7 prefectures, and 3 leagues. In order to ensure the integrity of the map, 30 counties directly under province are also among them. Hong Kong, Macao, and Taiwan of China were not included in the research scope.

### 2.2. Data

The Nighttime light image used in this paper was acquired by the Visible Infrared Imaging Radiometer Suite (NPP-VIIRS) on the National Polar-orbiting Satellite of the United States, The Earth Observation Group (EOG) compiled by VIIRS nighttime lights (VNL) V2. (https://eogdata.mines.edu/products/vnl/, accessed on 10 March 2022). This data filtering removes cloudy, sunny, and moonlight exposures, removes high and low emissivity outliers and biomass burning pixels, and separates the background. The spatial distribution of population in 2020 was acquired by WorldPop (http://www.worldpop.org.uk, accessed on 10 March 2022). The data mainly use Landsat Images to identify residential areas. Next, Random Forest estimation was used to generate regional population data [24]. The spatial resolution was 1 km. The GDP spatial distribution data were acquired by the Resource and Environment Science and Data Center (https://www.resdc.cn/Default.aspx, accessed on 10 March 2022) of CAS, with a spatial resolution of 1km. The Digital Elevation Model (DEM) acquired by the United States Geological Survey (https://lta.cr.usgs.gov/HYDRO1K/, accessed on 10 March 2022) had a spatial resolution of 90 m. Slope analysis and zoning statistics were used to count the elevation, average slope, and topographic fragmentation of each prefecture-level city in China. All kinds of spatial data were re-projected to the CGCS2000 coordinate system and re-sampled to 1 km resolution.

The Point of Interest (POI) data were obtained from the Amap platform, and the nationwide POI data for 2020 were obtained through operations such as correction, rectifying, and spatial matching (Table 1). The third space refers to people’s shopping and leisure places, including five POI categories: catering service, shopping service, life service, sports and leisure service, and scenic spots. The POI data were superimposed with the administrative boundary vector map or grid to calculate the information entropy of each unit. The ratio of the number of third spatial POI to the area in each unit was counted as the third spatial density index value. China’s traffic road data in 2020 were obtained from Amap, including railways (3259 records of railway network), expressways (261,607 in total), national roads (333,629 in total), provincial roads (622,148 in total), county roads (453,943 in total), and township roads (453,943 in total). By referring to Fan et al.’s definition of the capacity of different road grades, the weight of different roads is determined [25].

The administrative boundary or grid is used to divide the road data at all levels, the density of the road network at all levels in the unit is counted, and the weighted overlay is used to obtain the traffic degree index value. Baidu index is a data sharing platform that is based on Baidu’s massive user behavior data. In this paper, “online activities” and “offline activities” were used as keywords to search the overall daily mean of all cities in China in 2020 as the word frequency of cultural and creative activities.

In 2020, Tencent’s social activity, the number of Talkingdata active devices, Tiktok users and activity, number of bars, night travel activity, Tiktok night check-ins, number of Livehouses, night movie activity, and urban public transport activity at night were obtained from the CBN·New First-tier Knowledge City data platform. The history and culture, social security, social equity, housing cost, openness, health care data, etc., of cities were obtained from the Numbeo database and the Amap platform, which together constitute the index of social inclusion. The data on the Didi platform were obtained from the last city where the user was located during the period after the Spring Festival (from the 18th to the 22nd day of the first lunar month); this flowed into cities after the Spring Festival, and the ratio of Didi orders at night to the whole day orders was obtained.

Data on the growth of the permanent population, the degree of the youth of the population, and the quality of the population from 2010 to 2020 are from the Seventh National Census Bulletin of the whole country and all provinces. Data such as patent applications, employment data, and the proportion of tertiary industries were obtained from the official websites of the National Bureau of Statistics, provincial and municipal statistical departments, as well as from the China Urban Statistical Yearbook and provincial and municipal statistical yearbooks. The administrative divisions are from the National Geomatics Center of China (http://www.ngcc.cn/, accessed on 10 March 2022), and the base map has not been modified.

Due to the unavailability of data and different statistical caliber, Hong Kong, Macao, and Taiwan were not included in the scope of the study. The spatial scope of the urban agglomeration comes from the urban agglomeration development plan approved by the National Development and Reform Commission of China.

### 2.3. Methods

#### 2.3.1. Urban Vitality Measurement

**Figure 1:**
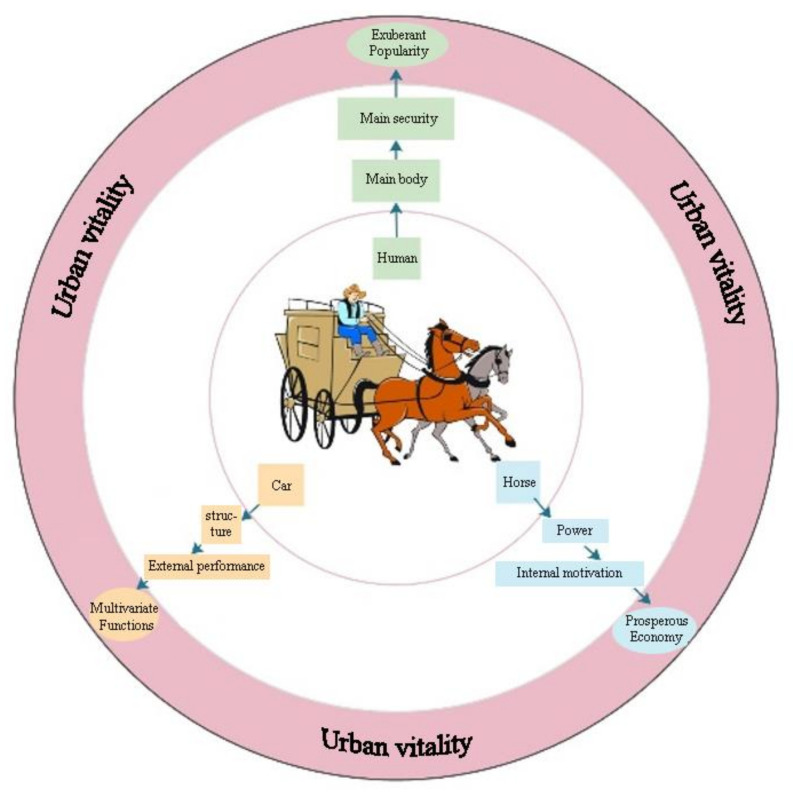
Mapping scheme of urban vitality.

On the basis of reference to previous studies [10,14,18,26,27,28], this paper holds that urban vitality refers to the exuberant vitality of a city, which reflects the endogenous force of urban development, and is the comprehensive ability of a city to maintain normal and active operation, which is manifested as exuberant popularity, prosperous economy, and multivariate functions. If the city is compared to a horse-drawn carriage, then people, cars, and horses represent three aspects of urban vitality (Figure 1). As the main body of the carriage, people are the main guarantee for the normal running of a carriage, which is reflected in the vitality of the city; an exuberant popularity is the main guarantee for a dynamic city. The horse is the driving force of the carriage, which is reflected in the vitality of the city. Economic affluence can reflect the inner motivation of a dynamic city. The cart is the structure of a horse-drawn carriage, reflecting the external expression of a horse-drawn carriage and mapping to the vitality of the city; multivariate functions can reflect the external expression of a dynamic city.

The city is regarded as a complex multi-dimensional regional development synthesis, from the population, economy, and function of the three subsystems, we constructed an indicator system for the vitality of popular, economically affluent, and multi-functional cities (Table 2); the city’s individual and comprehensive vitality were measured at the grid, prefecture-level administrative, and urban agglomeration scales. Exuberant popularity is the main guarantee of urban vitality. The reasons for the selection of specific indicators are as follows: In terms of popularity, cities were born of human settlements, cities are dominated by people who live in them, and people are the core element of urban vitality, therefore, population density is an important index to measure urban vitality. Migration flows reflect the attractiveness of cities; a younger population structure will make the city more vibrant, and the quality of the population is also very important to characterize the vitality of a city. In terms of prosperous economy, the economic foundation is the solid backing for urban development; scientific and technological innovation is the inexhaustible driving force for a dynamic city.

The urban night economy is a new form of economic development, and the digital economy plays an increasingly important role in economic society. In terms of multivariate functions, the interaction between people and people in the physical space builds a diversified city, displays a diverse urban space, and makes the city more vibrant. Public activities are often accompanied by certain needs, namely city functions (accommodation, dining, work, shopping, etc.). An active city must maintain sufficient diversity to meet the individual needs of city residents. If the family space is called the first space, the workspace is called the second space, the space for leisure and entertainment can be called the third space, and the third space is the catalyst of urban vitality, which can most intuitively reflect the quality of life of a city. Cultural socializing involves how to provide quality spiritual services for the city. Social inclusion is a major feature of today’s cities. Urban transportation plays an important role in the flow of production factors; transportation provides the city with the flow of people, logistics, and other services, and promotes the vitality flow of the city.

It should be noted that the administrative region scale and urban agglomeration scale can only represent the overall urban vitality of a unit and cannot identify the vitality within the city, while the grid scale can measure the vitality of small units within the city and subdivide the spatial differences within the urban vitality. However, limited by data acquisition, not all indicators can generate spatial data, and some indicators must be deleted (Table 3). Although the indicators are smaller than the administrative scale, the main factors in the three dimensions of exuberant popularity, prosperous economy, and multivariate functions are not missing.

There will be differences in unit, order of magnitude, or trend among different indicators, so it is necessary to make the indicators dimensionless. According to the principle of non-deformation of distribution and variation characteristics, the maximum method is selected for dimensionless processing. The spatial distance model is used to calculate the urban vitality index, and the formula is as follows [29]:

(1) $$d_{i}=\sqrt{{\left(y_{i1}-y_{min\_1}\right)}^{2}+{\left(y_{i2}-y_{min\_2}\right)}^{2}+\cdots +{\left(y_{ij}-y_{min\_j}\right)}^{2}+\cdots +{\left(y_{in}-y_{min\_n}\right)}^{2}}$$

**Figure 2:**
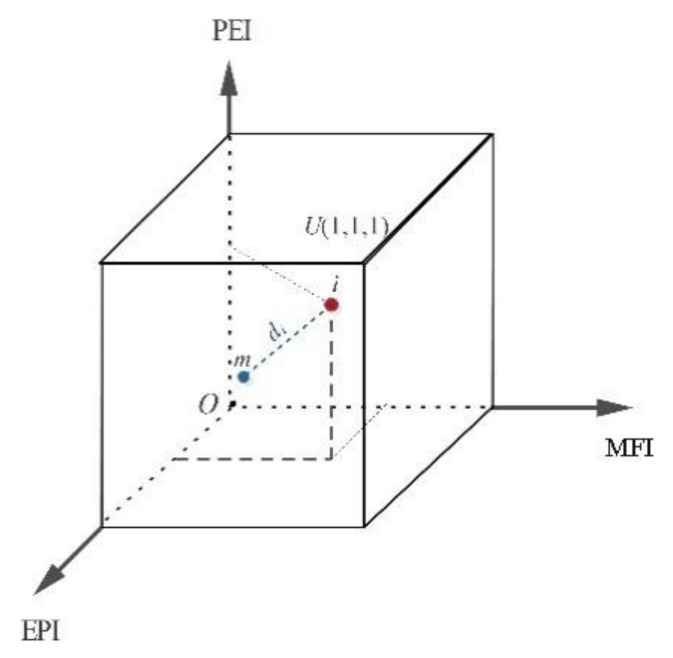
Concept map of SDM (As an example of measuring the UVI).

In this formula, *d_i_* is the comprehensive evaluation value of unit *i*, *y_ij_* is the dimensionless value of the *j*th index of the *i*th unit, *n* is the number of indicators, and *y_min_j_* is the minimum value of *j* indicators. The spatial distance model based on Euclidean distance does not need to define the weights artificially; it has the advantages of a simple algorithm and clear mechanism, and the evaluation result is objective. Figure 2 takes the three indexes to synthesize the city vitality composite index as an example. Firstly, three-dimensional space is established to determine the lowest point m (the distance *d_i_* of the lowest point m is used as the comprehensive vitality index of research unit *i.* The larger *d_i_* is, the more active the city it is; *U* is the highest point, and the distance *d_U_* between the highest value and the lowest value is the maximum value of the comprehensive vitality index of the city).

Exuberant popularity is the main guarantee of urban vitality. People are the core element of urban vitality, so population density is an important indicator to measure urban vitality. With the continuous improvement of China’s transportation facilities, people tend to move to more attractive cities, so population attraction is an important reference for cities to have an advanced and superior nature. Cities will become more dynamic because of their younger demographic structure. The level of population quality is very important to characterize urban vitality. Therefore, Exuberant Popularity Index (EPI) was obtained by weighted sum using the above indexes.

Economic foundation is the solid backing of urban development and an important representation of urban vitality. Per capita GDP can reflect the overall level of economic development. Scientific and technological innovation is the inexhaustible driving force for a dynamic urban development. The urban night economy can stimulate the consumption level of residents. Digital economy is gradually playing an increasingly important role in the economy and society. Therefore, the Prosperous Economy Index (PEI) was obtained by weighted summing using the above indexes.

Diversification of urban functions is the source of urban vitality. An active city must maintain enough diversity to meet the personalized needs of urban residents. The third space is the catalyst for urban vitality, which intuitively reflects the quality of life of a city. Cultural socialization involves how to provide spiritual quality services to cities. Social inclusion is a major feature of today’s cities. The traffic of the city promotes the flow of the vitality of cities. Therefore, the Multivariate Functions Index (MFI) was obtained by weighted sum using the above indexes.

#### 2.3.2. Space Scanning

Dynamic cities with high development potential were detected by spatial scanning metrology, and potential clusters that need to be focused on in the future were further identified. Nonrandom geographic processes make spatial clusters, and the identification of spatial clusters is an important problem in spatial science. Space scanning uses LLR (Log Likelihood Ratio Test Statistic) to identify spatial clusters of a geographical phenomenon, and it can not only detect whether there is agglomeration in geographical space, but also determine the location and risk level of the spatial cluster [30,31]. Common spatial scanning methods include SaTScan and Flexible; the SaTScan’s scan window is a circle or ellipse, but Flexible’s scan window is irregular. Considering that prefecture-level administrative districts are not standard circles or ellipses, and their boundaries are irregular, this paper chooses the Flexible method and uses the FleXScan software for cluster identification. Firstly, the theoretical value of urban vitality index is calculated according to Poisson distribution. Secondly, the actual value and theoretical value of urban vitality index are used to construct LLR to evaluate the prominent aggregation degree of the urban vitality within the scanning window. The larger the LLR, the higher the urban vitality contained in this window. Finally, the scanning window of the maximum LLR is regarded as the active city cluster, the cities contained in the cluster are retrieved, and the relative risk degree of the cluster is calculated, and the significance level of LLR (*p* value) is calculated by Monte Carlo Method.

In all scan windows *Z* in the study area, *SS* denotes the maximum likelihood ratio:

(2) $$SS=\frac{\frac{max}{Z\left\{L\left(Z\right)\right\}}}{L_{0}}=\frac{max}{Z}\left\{\frac{L\left(Z\right)}{L_{0}}\right\}$$

In the formula, *L*(*Z*) is the log-likelihood ratio of scan window *Z*, and *L*_0_ is the log-likelihood ratio under invalid hypothesis. *LLR* is as follows:

(3) $$LLR=\frac{L\left(Z\right)}{L_{0}}=\frac{{\left[\frac{n\left(Z\right)}{\mu \left(Z\right)}\right]}^{n\left(Z\right)}{\left[\frac{N-n\left(Z\right)}{\mu \left(N\right)-\mu \left(Z\right)}\right]}^{n\left(N\right)-n\left(Z\right)}}{{\left[\frac{N}{\mu \left(N\right)}\right]}^{n\left(N\right)}}$$

In the formula, *n*(*Z*) and *μ*(*Z*) respectively represent the actual and theoretical values of the urban vitality index in the scanning window *Z*, *N* and *μ*(*N*) respectively represent the actual value of the national urban vitality index and the theoretical value under the invalid hypothesis, *μ*(*N*) = *N*.

### 2.4. Multi-Scale Geographical Weighted Regression

Although it can deal with spatial heterogeneity problems, Geographically Weighted Regression (GWR), according to the data of spatial attribute weighted regression (with all the independent variables having the same spatial scale (bandwidth)), may cause the regression results to be unsound [32]. Multi-scale Geographically Weighted Regression (MGWR) overcomes this problem. MGWR supports that each independent variable has an appropriate and independent bandwidth, and the bandwidth of the variable can reflect the scale of the spatial effect of the variable on the dependent variable; the spatial process model constructed by using the multi-bandwidth method is more effective and real [33]. Urban vitality is a complex and multi-dimensional urban index, which is the result of the joint action of many factors. To explore its influencing factors, the influence scale of independent variables should be fully considered in order to avoid the instability of the model due to the consistent size of the independent variables, thus affecting the analysis results. In this paper, the MGWR model was adopted to explore the difference in the impact scale between independent variables and urban vitality, and compared with the results of the classical GWR model, a better model was selected to analyze the main influencing factors of urban vitality.

(4) $$\mathrm{GWR}:y_{i}=\overset{k}{\underset{j=1}{\sum }}\beta_{k}\left(\mu_{i},\nu_{i}\right)x_{ij}+\varepsilon_{i}$$

In the formula, *β_k_* is the regression coefficient obtained by local regression.

(5) $$\mathrm{MGWR}:y_{i}=\overset{k}{\underset{j=1}{\sum }}\beta_{bwj}\left(\mu_{i},\nu_{i}\right)x_{ij}+\varepsilon_{i}$$

In the formula, *bwj* is the bandwidth determined by the *J*th *variable. β_bwj_* is the regression coefficient obtained by local regression. The window size of local regression is controlled by the bandwidth *bwj*. The kernel function and bandwidth selection of MGWR still follow the method of the classical GWR model, and the common quadratic kernel function and AICc criterion are selected in this paper.

## 3. Results

### 3.1. Spatial Pattern of Urban Vitality in China

#### 3.1.1. Characteristics of Urban Vitality at Grid Scale

**Figure 3:**
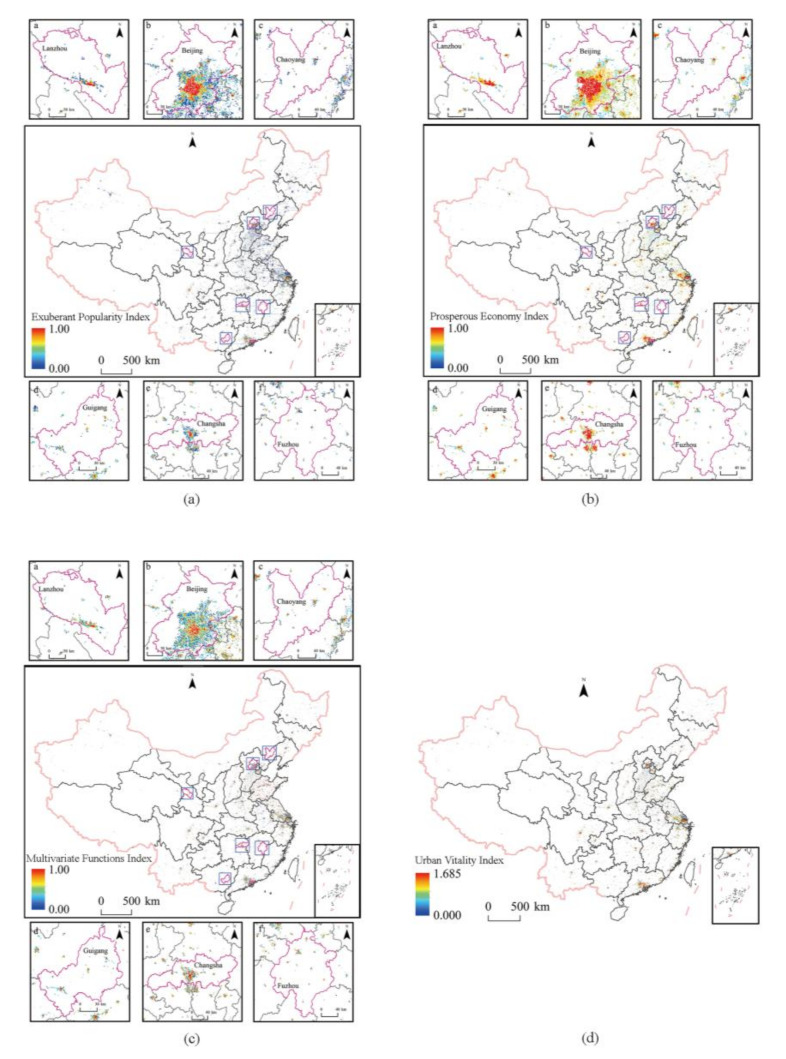
Spatial distribution of UVI of urban areas in China at grid scale: (**a**) Exuberant Popularity Index, (**b**) Prosperous Economy Index, (**c**) Multivariate Functions Index, and (**d**) Urban Vitality Index. a: Lanzhou City, b: Beijing City, c: Chaoyang City, d: Guigang City, e: Changsha City, f: Fuzhou City.

**Figure 4:**
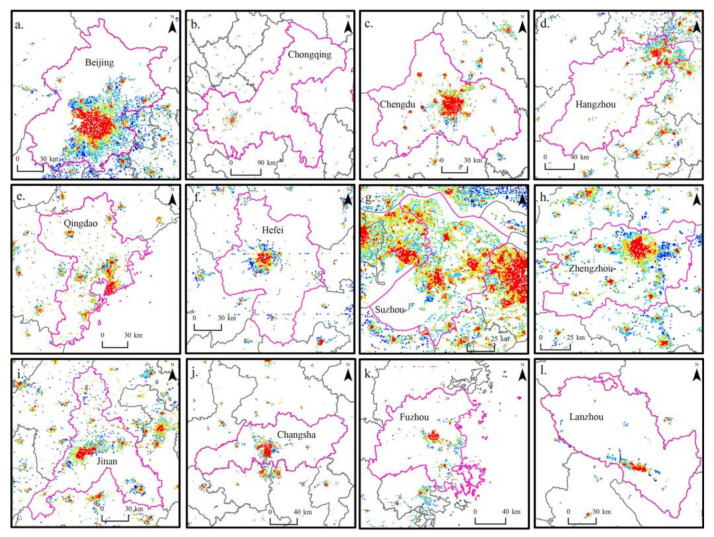
Spatial distribution of UVI of some cities on grid scale: (**a**) Beijing City, (**b**) Chongqing City, (**c**) Chengdu City, (**d**) Hangzhou City, (**e**) Qingdao City, (**f**) Hefei City, (**g**) Suzhou City, (**h**) Zhengzhou City, (**i**) Jinan City, (**j**) Changsha City, (**k**) Fuzhou City, and (**l**) Lanzhou City.

The Exuberant Popularity Index (EPI), Prosperous Economy Index (PEI), Multivariate Functions Index (MFI), and Urban Vitality Index (UVI) were calculated on the grid scale in China in 2020 to obtain the spatial distribution of urban vitality on the grid scale (Figure 3). Limited by the size of the illustrated edition, the spatial distribution of the vitality of each subsystem cannot be clearly analyzed from the small and medium-sized regional scale in the paper. Therefore, six representative cities such as Lanzhou, Beijing, Chaoyang, Guigang, Changsha, and Fuzhou (In Jiangxi Province) are selected for display. These cities can reflect the differences between north and south, east and west, plain and valley cities, as well as the differences in city size. In addition, some representative city maps are selected for display (Figure 4). In Figure 4, Fuzhou is located in Fujian Province. In China, “Fuzhou” refers to two cities, so we mark them in the paper for the convenience of distinction.

Overall, the spatial distribution of urban popularity exuberance at the grid scale (Figure 3a) is dominated by medium and low levels (blue and green areas in the figure), showing a trend of high southeast and low northwest. The regions with high EPI were mainly distributed on the southeast side of Hu Huanyong Line (“Hu Huanyong Line” is a comparison line for dividing China’s population density proposed by Chinese geographer Hu Huanyong in 1935; geographically, the line starts in Heihe city, Heilongjiang Province, and ends in Tengchong city, Yunnan Province), especially in North China, East China, and the Greater Bay Area. Provincial capital cities and their surrounding urban areas were also the regions with high EPI. The areas with high PEI (Figure 3b) were mainly concentrated in the coastal areas of North, East, and South China, especially in the Beijing–Tianjin–Hebei region, and Shandong Peninsula. The areas with high MFI (Figure 3c) were arranged radially around each provincial capital. The spatial distribution of urban vitality shows a pattern of “large dispersion and small aggregation” (Figure 3d). The areas with high vitality are concentrated in the Yangtze River Delta, the Beijing–Tianjin–Hebei region, and the Greater Bay Area, and scattered in and around urban centers (cities and counties). Urban vitality has obvious differentiation characteristics of city scale and grade directivity. The areas with high vitality in the provincial capital and above cities have a wide range and can drive the development of the surrounding cities to form a patchy whole with high vitality. It is worth noting that most of the surrounding areas near the central city have lower vitality than those far away from the central city. This may be due to the fact that the siphoning effect is more pronounced closer to the city, the central city is more attractive, and thus the regional vitality level close to the central city is lower.

#### 3.1.2. Characteristics of Urban Vitality at Prefecture-Level Administrative

**Figure 5:**
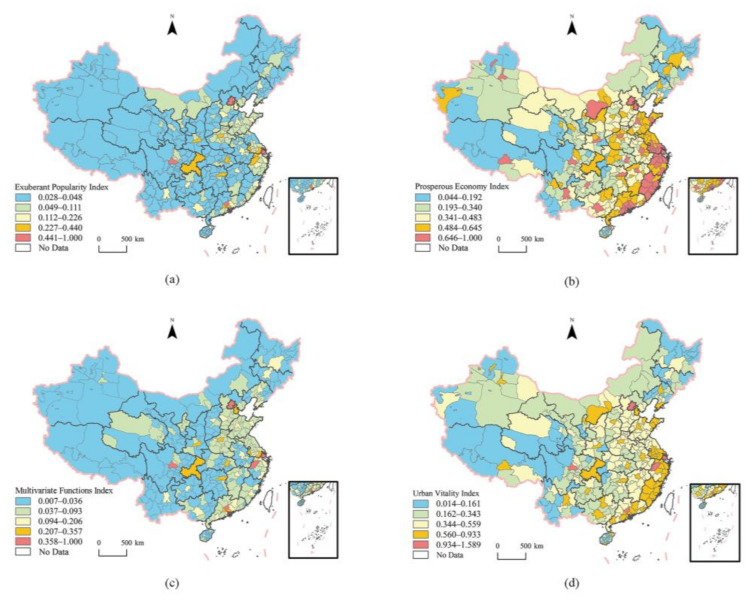
Spatial distribution of urban vitality index of Chinese cities: (**a**) Exuberant Popularity Index, (**b**) Prosperous Economy Index, (**c**) Multivariate Functions Index, and (**d**) Urban Vitality Index.

Taking 367 prefecture-level and above cities in China as the evaluation object, the EPI, PEI, MFI, and UVI of prefecture-level administrative regions in 2020 were calculated, and the spatial distribution of each vitality index was obtained (Figure 5). The value of vitality index was divided into five grades by the natural break point method, which were low, low, medium, high, and high from low to high. Table 4 shows the subregional statistics of the four categories of indicators.

Figure 5a and Table 4 show that the average EPI of cities at prefecture-level and above in China is 0.074, indicating that the overall popularity is relatively low. There are significant regional differences in EPI, showing the highest spatial distribution in the east, followed by the middle and west, and the lowest in the northeast. The degree of dispersion among cities in the east is the highest, and the development is uneven. As far as south and north are concerned, the population exuberance of southern cities is greater than that of northern cities. The most popular regions are mainly in Zhejiang, Guangdong, Shandong, Henan, and Jiangsu, which coincide with the provinces with the largest population growth in the Seven National Census Data. Shenzhen, Beijing, Guangzhou, Chengdu, and Shanghai ranked the highest rank, EPI belongs to a higher level of the city of Xi’an, Dongguan, Zhengzhou, Hangzhou, Nanjing, Chongqing, Changsha, Huzhou, Suzhou, Foshan, Xiamen, and Wuhan. In addition, Chengdu, Xi’an, and Chongqing were shortlisted and are all distributed in the east, with no central cities being shortlisted. The EPI of cities with provincial capitals and above was 3.7 times the national average.

Figure 5b shows that the urban PEI of China, overall, presents a spatial distribution trend of gradually decreasing from the southeast coastal areas to the inland areas, and forms an oblique “n” structure with the Shandong Peninsula–Chongqing, Shandong Peninsula–Yangtze River Delta, and Yangtze River Delta–Greater Bay Area as the basic skeleton. Overall, the PEI is East > Central > West > Northeast, South > North China. Unlike the EPI, the PEI is more evenly distributed across cities in each class. There are 51 cities in the top, and the top 10 cities are Shenzhen, Guangzhou, Beijing, Shanghai, Zhuhai, Suzhou, Yichun, Hangzhou, Wuxi, and Karamay. From the numerical point of view, the average value of the economic affluence index is 0.396, which is in the middle level, and the standard deviation is 0.210. The development difference between cities is large. It is worth mentioning that the difference between cities above the provincial capital level is smaller than that between cities in China.

As shown in Figure 5c, the MFI of Chinese cities, overall, shows a moderate to lower level, with an average value of 0.060, ranking in the second rank. The spatial distribution shows a trend of East > Central > Northeast > West China, and the functional diversity degree of southern cities is greater than that of northern cities. Beijing, Shanghai, Guangzhou, Shenzhen, Hangzhou, and Chengdu were ranked high on the MFI, while Chongqing, Suzhou, Nanjing, Wuhan, Foshan, Changsha, Zhengzhou, Dongguan, Tianjin, and Xi’an were not so high.

Figure 5d shows that the UVI of urban vitality in China shows a spatial distribution trend with a high value in the southeast and a low value in the northeast and northwest regions, with an average value of 0.368, ranking in the third level, indicating that the overall vitality degree of Chinese cities is not high. The number of cities with low grades accounted for about 48.5%. There were 61 cities with high grade, and only Beijing, Shenzhen, Shanghai, Guangzhou, Chengdu and Hangzhou were in high grade. In the western region, except Chengdu, Xi’an, Karamay, Ordos, Chongqing, Kunming, Nanning, Urumqi, Guiyang, Lhasa, Liuzhou, Hohhot, Yinchuan and other cities have relatively high vitality, while in the northeast region, except Dalian and Shenyang, other cities are in the low rank.

**Figure 6:**
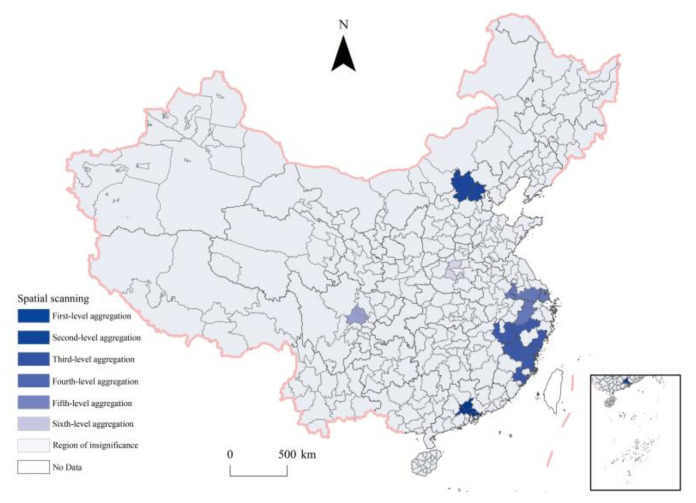
Cluster distribution of urban vitality index based on Flexible spatial scan statistics.

The global Moran’s I value of China’s UVI calculated by GeoDA software was 0.437, which passed the significance test of 0.01, indicating that the UVI at the prefecture level had significant spatial autocorrelation characteristics. Spatial scanning detection was used to detect six potential clusters that passed the 0.01 significance test (Figure 6), which were divided into six grades according to LLR from high to low. The LLR of the first-level aggregation area is 4.118, covering Guangzhou, Shenzhen, Dongguan and Foshan. The LLR of the second-level aggregation area was 1.978, covering Beijing and Zhangjiakou. The LLR of the third-level aggregation area was 1.710, covering Fuzhou, Ningde, Nanping, Quanzhou, Shangrao, Yingtan, Jinhua, Quzhou, and Wenzhou. The LLR of the fourth-level aggregation area was 0.736, covering Shanghai, Ma’ anshan, Nanjing, Changzhou, Suzhou, Wuxi, Hangzhou, and Huzhou. The LLR of the fifth-level aggregation area was 0.536, covering Chengdu and Deyang. The LLR of the sixth-level aggregation area was 0.337, covering Zhengzhou, Luohe, and Pingdingshan. In the future, we should pay more attention to these six vitality clusters to drive the vitality growth of surrounding cities and form a broader and more concentrated vitality cluster.

#### 3.1.3. The Vitality of Urban Agglomeration

**Figure 7:**
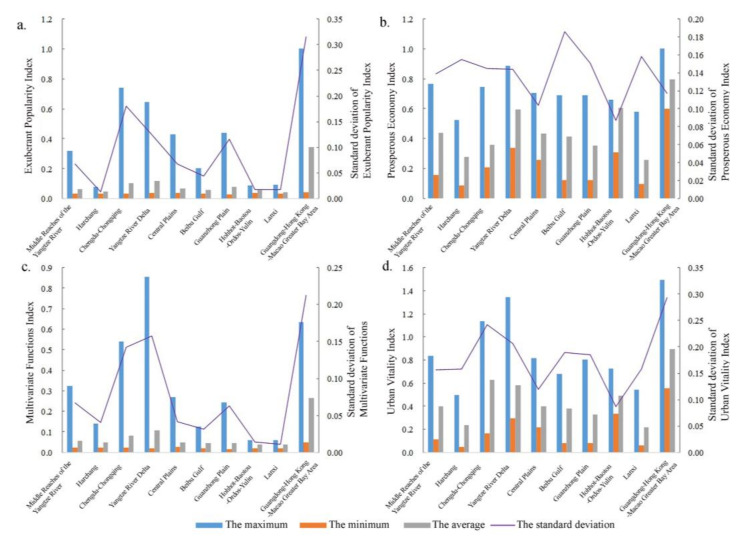
The vitality of each dimension of state-level Urban Agglomerations: (**a**) Exuberant Popularity Index, (**b**) Prosperous Economy Index, (**c**) Multivariate Functions Index, and (**d**) Urban Vitality Index.

An urban agglomeration is the main platform that supporting China’s high-quality development and the main spatial form of promoting the country’s new urbanization. In this paper, 10 national-level urban agglomerations approved by The State Council are selected to calculate their vitality, and the results are shown in Figure 7. From high to low, the UVI of the urban agglomeration was Guangdong–Hong Kong–Macao Greater Bay Area > Chengdu–Chongqing > Yangtze River Delta > Hohhot–Baotou–Ordos–Yulin > Middle reaches of the Yangtze River > Central Plains > Beibu Gulf > Guanzhong Plain > Harchang > Lanxi Urban agglomeration. The differences among cities within the urban agglomerations are as follows: Guangdong–Hong Kong–Macao Greater Bay Area > Chengdu–Chongqing > Yangtze River Delta > Beibu Gulf > Guanzhong Plain > Lanxi > Harchang > Middle Reaches of the Yangtze River > Central Plains > Hohhot–Baotou–Ordos–Yulin urban agglomeration. Based on the measurement results of the three-dimensional indexes and the UVI, the urban agglomeration can be divided into three gradients. The Guangdong–Hong Kong–Macao Greater Bay Area, Yangtze River Delta urban agglomeration, and Chengdu–Chongqing urban agglomeration are the first gradients. In this gradient, an urban agglomeration is prominent in the three dimensions of popularity, economy, and function, as well as the comprehensive development of urban vitality, and the urban agglomeration is full of vitality. The second gradient includes Hohhot–Baotou–Ordos–Yulin, the middle reaches of the Yangtze River, the Central Plains, and Guanzhong Plain. The urban agglomeration of this gradient has a good development situation, but there are some development shortcomings and there is still a gap with the first gradient. Beibu Gulf, Harchang, and Lanxi urban agglomerations are the third gradients, which needs to enhance the vitality value.

### 3.2. Influence Mechanism of Urban Vitality

#### 3.2.1. Influencing Factors of Urban Vitality

Urban vitality is influenced by many factors. Referring to previous studies [18,28,34,35], this paper argues that the influence factors of urban vitality can be divided into the internal characteristics and the external environment, including the natural conditions, environment quality, public services, industrial economy, space form, policy guidance, and the influence of various factors such as the size of the location. The influence factors were selected to construct the index system. After the collinearity test, the index system composed of 18 evaluation indexes was obtained after the index with a coefficient of variance expansion greater than 7.426 was eliminated (Table 5). Limited by data acquisition, 291 cities with comprehensive data were selected for analysis. Because there may be large topographic fluctuation within the city, and the average method will produce deviation, the mode of elevation within the city is used to represent the overall elevation of the city. The degree of terrain fragmentation is expressed by the standard deviation of urban elevation points. The compactness of construction land is based on the Richardson compactness model [36]. The intensity of urban development and utilization is the ratio of the built-up area to the land area of the administrative area, and the utilization rate is the ratio of the urban construction land area to the built-up area. The city size is the nominal data, and the value of first-tier cities, new first-tier cities, second-tier cities, third-tier cities, fourth-tier cities, and fifth-tier cities are assigned from 6–1 according to the 2020 City Rank Ranking of New First-Tier Cities Research Institute of China Business Network.

Using quadratic kernel function and AICc criterion, MGWR and GWR models of urban vitality and each influencing factor were constructed. The fitting effects of the Global Regression model (GR), GWR, and MGWR were compared (Table 6). The RSS and AICc of the MGWR model are significantly lower than those of the other two models, and the goodness of fit R2 is the highest, which effectively improves the robustness of the model. Compared with the classical GWR model, the MGWR model considers the action scale of independent variables, which provides support for exploring the spatial effect of each influencing factor.

MGWR can directly obtain the scale of differentiation of each variable, while GWR can only reflect the single and undifferentiated scale of all variables. The bandwidth of GWR was 117, accounting for 40.2% of the total number of samples. The MGWR scale analysis results showed that the bandwidth of different variables was significantly different. The action scale of per capita domestic electricity consumption is very small, with a value of 43, which is lower than the action scale of other variables, indicating that its spatial heterogeneity is obvious. The action scale of scientific expenditure is 65, the intensity of urban development and utilization is 75, the distance from the nearest city above the provincial capital is 79, the balance of household savings is 83, and the proportion of the secondary industry and the proportion of the tertiary industry is 90. These belong to a smaller scale. The action scale of participation in endowment insurance is 104, accounting for 35.7% of the total sample; the action scale of total import and export is 96; the action scale of public financial expenditure is 101. They belong to a larger scale. The coefficients of these indicators are relatively stable in space. The action scale of annual average PM_2.5_ concentration is 290, which is a global scale. There is almost no spatial heterogeneity. Urban vitality is affected by this factor in almost the same way.

**Figure 8:**
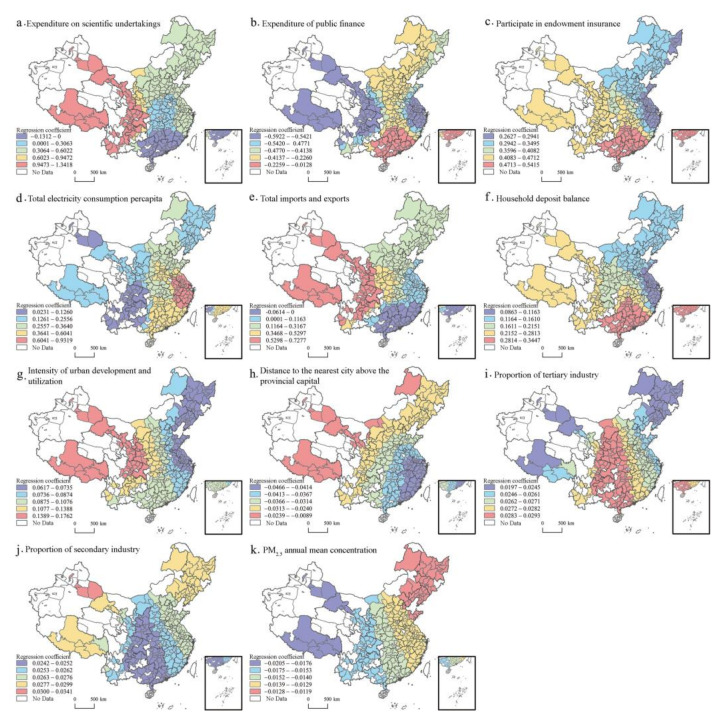
Spatial distribution of regression coefficients in the MGWR.

The spatial distribution map of regression coefficients of 11 urban vitality influencing factors with a small bandwidth and significant global regression coefficients (Figure 8) was made to explore the spatial differences of their impacts on urban vitality. Urban vitality is most influenced by policy orientation. Figure 8 shows that the regression coefficient of scientific expenditure is positive, indicating that scientific research investment will improve urban vitality. The regression coefficient of public financial expenditure is negative, and the absolute value of the coefficient is low in the south and high in the north. The coefficients of the proportion of public services and participation in pension insurance are positive, and the regression coefficient of the per capita total domestic electricity consumption is positive, and East China > South China > Central China > North China > Northeast > Northwest > Southwest. Urban vitality is positively affected by the industrial economy, indicating that the improvement of the industrial economy contributes to urban vitality. The regression coefficients of total import and export are mostly positive and highest in the western region. The growth of household deposit balance is conducive to the construction of urban vitality. The regression coefficient decreases gradually from the center of South China to the surrounding areas. In terms of urban spatial form, the only influence factor that has passed the significance test is the intensity of urban development and utilization, the coefficient of which is positive and gradually increases from east to west. The distance from the nearest city above the provincial capital has a negative impact on the city vitality, and the absolute value of the regression coefficient is higher in the southeast and lower in the northwest. The annual average concentration of PM_2.5_ also has a negative effect on urban vitality.

#### 3.2.2. The Influence Mechanism of Spatial Difference of Urban Vitality

**Figure 9:**
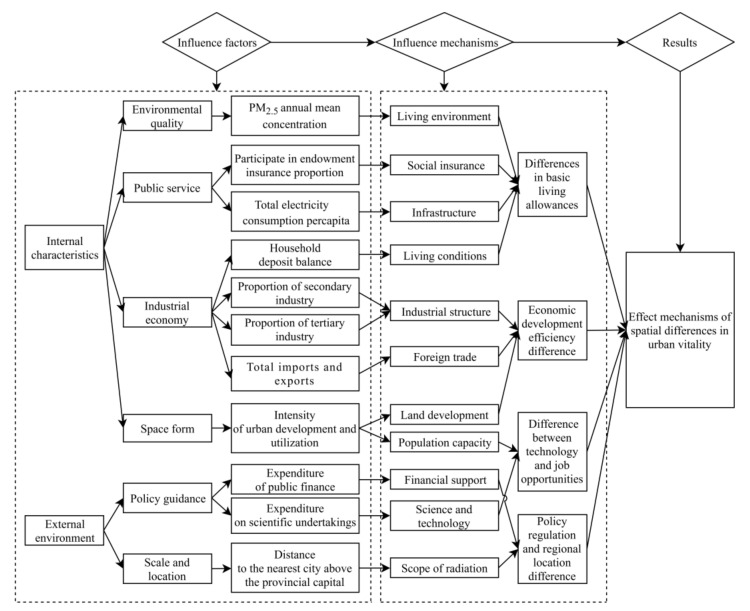
Influential mechanisms of spatial differences in urban vitality in China.

The ultimate purpose of building urban vitality is to meet people’s demand for a better life. Based on the results obtained by MGWR and with reference to previous studies [37,38,39], the influencing mechanism of urban vitality difference in China is summarized (Figure 9). The construction of urban vitality is a complex process of population, economy, and function. It is influenced by internal characteristics and external environment, including environmental quality, public service, industrial economy, spatial form, policy orientation, scale location, and other factors. It can be seen from the above that among the driving factors affecting the difference in urban vitality, the annual average concentration of PM_2.5_, the proportion of pension insurance, the total per capita domestic electricity consumption, and the household deposit balance respectively represent the living environment, social security, infrastructure equipment, and living conditions, providing basic guarantees for life.

The basic quality of life and the convenience of basic services directly affect the mobility and living behavior of the urban population. The higher the basic guarantee of life, the more attractive the city is to the population, and the higher the level of urban vitality, which plays a fundamental role in supporting urban vitality. The proportion of secondary industry and tertiary industry reflects the industrial structure. Total imports and exports reflect foreign trade. Urban land development and utilization intensity reflect the degree of land development. Together, they constitute the industrial development and layout of the city. The agglomeration of resources, industries, and other factors can promote the city to become the growth pole of regional economic and social development. Industrialization is an important driving force for urban development, the service industry is booming, and the industrial structure is advanced and modernized. Foreign trade plays a role in boosting the city economy.

The better-developed cities are, the higher the degree of land development and the more prominent the development. Therefore, the efficiency of economic development is the direct drive to create urban vitality. Of course, urban development also represents a city’s population capacity. It provides space for human activity and it also reflects the potential to attract people to live and the labor force to work. The promotion of science and technology can regulate the vitality of cities. Financial support and the degree of radiation from the central city are the inducing factors of urban vitality. The geographical location represents the degree of connectivity between a city and neighboring central cities. Superior geographical location is more conducive to the flow of various elements between regions. Policy regulation and location factors promote or hinder the construction of urban vitality as external conditions.

## 4. Discussion

### 4.1. Comparison with Similar Research

In examining the relationship between multi-dimensional urban form and urban dynamism at the street level, Xia et al. [27] explores their changes in 15 megacities in China and identifies the successes and failures of good urban form norms. In their conclusion, the urban vitality index in prosperous areas is relatively high, while that in areas with weak economy has low vitality index. Further, the spatial agglomeration degree of different urban agglomerations is also different. In our study, the prosperous economy index in China as a whole is gradually decreasing from the southeast coastal areas to the inland areas. Similar spatial distribution characteristics of vitality in urban agglomerations also exist, and there are obvious spatial differences in urban vitality characteristics within different urban agglomerations. Most current studies have taken small areas (neighborhoods) or single urban areas as the research scale. In terms of data sources, most studies use POI data in combination with other cultural and economic data. In a multi-dimensional urban vitality measurement of streets in Chengdu, Han et al. [40] uses taxi and bike-sharing tracks and POI data to identify the spatial pattern of urban vitality from social, economic, and cultural dimensions. The study has found that regions with high economic and cultural dynamism tend to have high urban dynamism; our study also presented a similar view. Liu et al. [41] evaluated the influencing factors of urban vitality using POI data, combined with mobile phone signaling data and communication base station range data. It concludes that there is spatial heterogeneity in the spatial pattern and drivers of urban vitality both economically and socially. After introducing more indicators and exploring them, we found significant spatial heterogeneity in the total per capita domestic electricity consumption in the selected index, and this indicated that the action scale of this influencing factor is small. Pan et al. [42] conducted an analysis of urban vitality indicators and distribution patterns using points of interest and microblog check-in geotags. Their study suggests that point-of-interest and social media check-in data can be a better indicator of urban vitality.

In addition, more data characterizing urban vitality were also included, such as the population quality level, the technological innovation competitiveness, the digital economy index, and the word frequency of cultural and creative activities. More comprehensive POI data will play a positive role in the assessment of urban vitality. Zikirya et al. [43] innovatively combined their research on urban vitality with urban takeaway, and explored the relationship between urban vitality and urban takeaway distribution. In their study, there was a significant spatial correlation between urban takeout vitality and urban vitality, but different cities had different correlation in different periods. Kim [44] explored the influence of urban form and land use on the vitality of urban areas within the geographical boundary. The result of his study implies the importance of highly desirable features for walking or transit-friendly neighborhoods. This kind of research starts from a certain branch and makes connections in the phenomenon of reflecting urban vitality. Comparing with their study, although the larger level and scale cannot better explain a more detailed aspect, they are helpful to explore the urban vitality at the macro level.

Zhou et al. [45] measured the spatial patterns, regional differences, and spatio-temporal evolution of urban green development efficiency (UGDE) in China from 2005 to 2015. They found that the Chinese UGDE showed a staged increase on the temporal scale. On the spatial scale, the UGDE had inverted pyramid cluster growth characteristics in different types of urban agglomeration. Morshed et al. [46] analyzed the urban transformation in Dhaka by using nighttime remote sensing data; they also found that although the regional size of Dhaka continues to grow with the growth of the population and the approach of the industrial center, it still dominates the newly generated urban hotspots in the surrounding urban clusters. This is somewhat similar to the conclusions of our study. On the administrative scale, the overall vitality degree of cities at the prefecture level and above in China is not high, the spatial difference is huge, and the degree of dispersion among cities is also high. On the scale of urban agglomerations, the vitality index of different urban agglomerations is obviously different, and the vitality among cities within the same urban agglomerations is also different.

In future studies, for the countries or regions with large spatial differences, a variety of spatial scales can be used to measure the urban vitality. At the same time, it is also necessary to obtain as many as possible highly accurate and diverse kinds of spatial data according to the local actual situation, which will help to accurately measure the vitality of the city. At present, most studies will choose the more common data to build the index system. How to find innovative data to conduct relevant research may become a potential direction.

### 4.2. Uncertainty and Limitation

Because of the complexity and multi-dimensional nature of urban vitality, the ambiguity and uncertainty of the concept. It causes great difficulties in the measurement of urban vitality. This paper considers the multi-dimension of urban vitality measurement, but it is biased. Limited by data acquisition and difficulty in spatialization, some socioeconomic raster data are difficult to obtain or prepare. The types of spatial data used in this paper are the highest resolution available for free, and the resolution can meet the requirements of displaying the spatial pattern in the whole country. However, the current resolution is still insufficient when studying the distribution of vitality within cities. In addition, there is no comparative study of multi-phase urban vitality measurement. The differences in vitality measurement methods and vitality creation mechanisms of other spatial scales (such as the scale of new cities) and long-term scales were not considered. In addition, due to missing data in individual regions, the results of the Moran’s I calculation may be affected.

In the future, we will try to focus on the long-term and multi-spatial scale research of urban vitality and compare the differences in spatio-temporal patterns and construction strategies of urban vitality under different scales, so as to provide reference for urban vitality creation and urban sustainable development. In the context of people-oriented urbanization, in the future, we can increase people’s subjective feelings and related indicators to jointly measure urban vitality. Questionnaires and other methods can be used to obtain people’s perception of all aspects of urban life, so as to realize the subjective and objective unity of measurement indicators and more accurately depict the vitality of cities.

### 4.3. Policy Suggestions

Based on the results of the study, we offer the following suggestions:

(1) We should make a reasonable plan for urban development. Through the study on the spatial distribution characteristics and spatial pattern of urban vitality, it is found that there is spatial differentiation of urban vitality, that its spatial difference is large, and the distribution is not balanced. When making city-related policies, we need to consider the city’s functional positioning and development basis. While promoting the flow of factors of production between cities, we also need to increase the links with central cities. We also need to de-emphasize the concept of city boundaries, which will help promote the interconnected development of urban clusters and strengthen inter-regional cooperation.
(2) We should optimize the environment in and around the city. Comfortable urban living environment will improve residents’ life satisfaction. At the same time, we need to optimize our infrastructure. Sound infrastructure is one of the important guarantees for building smart cities. These measures may improve the happiness of residents and thus effectively stimulate the vitality of the city.
(3) We need to promote the transformation and upgrading of the industrial structure. The new rational industrial structure will become an important driving force for urban development. At the same time, we also need to improve the efficiency of land use and pursue an appropriate increase in the size of the industry. These moves could contribute to a more hierarchical industrial layout.
(4) At the same time as technological innovation, we also need to keep the city with a “young” vitality and pay attention to the construction of cultural cities. We need a stronger sense of cultural belonging and a good education system. This will provide rich talent resources for urban development.

## 5. Conclusions

Different from other studies using general data and a single scale, this paper introduced a large number of big spatial data, based on three dimensions—exuberant popularity, prosperous economy, and multivariate functions from the grid—at the prefecture-level administrative region scale, and used the urban agglomeration of the three scales to build a vitality index system to measure the urban vitality. The MGWR model was used to explore the influencing factors of spatial difference in urban vitality. The main conclusions are as follows:

When analyzing urban vitality in refinement to the grid scale, the urban vitality presents a spatial distribution pattern of “large dispersion and small aggregation”, and it has obvious characteristics of city scale grade orientation. At the same time, cities with high vitality could play an obvious driving role in the surrounding cities with low vitality. At the prefecture-level administrative region scale, the overall vitality index of Chinese cities showed a high spatial distribution in the southeast, and a low spatial distribution in the northeast and northwest regions, with large spatial differences and a high degree of internal dispersion. At the urban agglomeration scale, there were three grades according to the degree of urban vitality. With the rise of grades, the vitality of urban agglomeration gradually decreases. In addition, the differences of vitality among cities within the same urban agglomeration are also different.

In this study, the influence mechanisms of urban vitality were influenced by a combination of internal characteristics and the external environment. Among the internal characteristics, quality of life of the population, convenience of basic services, and basic guarantee of life together play a supporting role in the vitality of the city. Industrial structure, foreign trade, and land development degree together constitute the industrial development layout. The aggregation of multiple factors such as industry and resources is the growth pole of regional economic and social development. Economic development efficiency is the direct driving force of urban vitality improvement. In the external environment, science and technology financial input will play a regulating role in the promotion of urban vitality to some extent. The superior geographical location of cities will be conducive to the communication of various elements between cities.

## Figures and Tables

**Table 1:** Data description.

Data	Spatial Resolution	Sources	Instruction
NPP-VIIRS	500 m	The Earth Observation Group (EOG) (https://eogdata.mines.edu/products/vnl/, accessed on 10 March 2022)	This data filtering removes cloudy, sunny, and moonlight exposures, removes high and low emissivity outliers and biomass burning pixels, and separates the background.
Spatial distribution of population data	1000 m	The WorldPop (http://www.worldpop.org.uk/, accessed on 10 March 2022)	Using Random Forest estimation to generate regional population data [24].
GDP spatial distribution data	1000 m	The Resource and Environment Science and Data Center of CAS (https://www.resdc.cn/Default.aspx/, accessed on 10 March 2022)	-
DEM	90 m	The United States Geological Survey (https://lta.cr.usgs.gov/HYDRO1K/, accessed on 10 March 2022)	Using slope analysis and zoning statistics to count the elevation, average slope, and topographic fragmentation of each prefecture-level city in China.
POI	-	The Amap platform (https://lbs.amap.com/, accessed on 10 March 2022)	The nationwide POI data for 2020 were obtained through operations such as correction, rectifying, and spatial matching.
China’s traffic road data	-	The Amap platform (https://lbs.amap.com/, accessed on 10 March 2022)	The administrative boundary or grid is used to divide the road data at all levels, the density of the road network at all levels in the unit is counted, and the weighted overlay is used to obtain the traffic degree index value.
Urban activity data	-	The CBN · New First-line Knowledge City data platform (https://www.datayicai.com/, accessed on 10 March 2022)	The data include Tencent’s social activity, number of Talkingdata active devices, Tiktok users and activity, etc.
Urban living data	-	The Numbeo database (https://www.numbeo.com/common/, accessed on 10 March 2022) The Amap platform (https://lbs.amap.com/, accessed on 10 March 2022)	The data include history and culture, social security, social equity, housing cost, etc.
Comprehensive population data	-	The Seven National Census Data	The data include patent applications, employment data, and the proportion of tertiary industries, etc.
Regional statistics data	-	The National Bureau of Statistics, provincial and municipal statistical departments The China Urban Statistical Yearbook, and provincial and municipal statistical yearbooks	-
Administrative divisions data	-	The National Geomatics Center of China (http://www.ngcc.cn/, accessed on 10 March 2022)	The base map has not been modified.

**Table 2:** Index system of urban vitality at the administrative region scale.

Dimension	First Indicators	Second Indicators	Calculation Method
Exuberant Popularity	Population to attract	Increase in permanent population	Annual increment of the permanent population in urban areas
		Attraction of population	Number of urban population inflows after the Spring Festival
	Population structure	Youth of the population	Percentage of population under 60
		Population quality level	Number of people with a college degree or above per 100,000
	Population accumulation	Population density	The ratio of permanent population to an area in an urban area
Prosperous Economy	Economic base	Per capita GDP	GDP in the downtown/Population
	Science and technology innovation	Technological innovation competitiveness	Comprehensive evaluation of patent applications, academic papers, scientific and technological enterprises, universities, and cultural facilities in the city
	Consumption scale	Night economy	Ratio of overnight Didi orders to full-day orders
	Digital information	Digital Economy Index	Comprehensive evaluation of digital and information construction, urban service, urban governance, and industrial integration in the city
Multivariate Functions	Versatile	POI information entropy	Information entropy of POI in urban area
	Social inclusion	Degree of social inclusion	Comprehensive evaluation of the city’s history and culture, social security, social equity, living cost, openness, and health
	Leisure entertainment	Third spatial density	Number and area ratio of shopping and leisure places in urban areas
	Social culture	Word frequency of cultural and creative activities	Average daily “online activity” and “offline activity” keyword search average
		Degree of social activity	Combined with Tencent social activity, the number of talkingdata active devices, TikTok users, and activity comprehensive evaluation
	Convenient transportation	Road traffic operation degree	Density of road network in urban areas

**Table 3:** Index system of urban vitality at grid scale.

Dimension	First Indicators	Second Indicators	Calculation Method	Spatial Data Sources
Exuberant Popularity	Population scale	Population density	Ratio of the human mouth and area in the grid	WorldPop data & Global city boundaries
	Population to attract	Population increment	Annual population increment in the grid	WorldPop data & Global city boundaries
Prosperous Economy	Economic base	GDP	GDP in the grid	GDP spatial grid data & Global city boundaries
	Night economy	Night light intensity	Ratio of the nighttime light intensity and area in the grid	Nighttime light data & Global city boundaries
Multivariate Functions	Function versatile	POI information entropy	POI information entropy in the grid	POI data & Global city boundaries
	Leisure entertainment	Third spatial density	Ratio of the number and area of shopping and leisure places in the grid	POI data & Global city boundaries
	Transportation convenient	Road traffic index	Grid road network density	National urban road data & Global city boundaries

**Table 4:** Statistics of four types of indexes by region and type.

Partition	EPI		PEI		MFI		UVI	
	AVG	STD	AVG	STD	AVG	STD	AVG	STD
East China	0.120	0.169	0.549	0.214	0.110	0.161	0.542	0.281
Central China	0.060	0.060	0.416	0.135	0.048	0.051	0.378	0.148
West China	0.055	0.077	0.311	0.189	0.036	0.057	0.275	0.204
Northeast China	0.046	0.019	0.255	0.143	0.042	0.033	0.215	0.146
North China	0.061	0.087	0.350	0.189	0.050	0.084	0.317	0.214
South China	0.084	0.126	0.434	0.219	0.069	0.112	0.410	0.259
Provincial capital and above	0.276	0.247	0.699	0.115	0.253	0.228	0.776	0.274
National	0.074	0.109	0.396	0.210	0.060	0.101	0.368	0.244

**Table 5:** Index system of influencing factors of urban vitality.

Type	Dimension	Evaluation Index	Unit
Internal characteristics	Natural conditions	Elevation *X*_1_	m
		Average slope *X*_2_	/
		Degree of terrain fragmentation *X*_3_	%
	Environmental quality	Green coverage rate *X*_4_	%
		PM_2.5_ annual mean concentration *X*_5_	ug/m^3^
	Public service	Participate in endowment insurance proportion *X*_6_	%
		Total electricity consumption per capita *X*_7_	10,000 kwh
	Industrial economy	Proportion of secondary industry *X*_8_	%
		Proportion of tertiary industry *X*_9_	%
		Total imports and exports *X*_10_	10,000 yuan
		Household deposit balance *X*_11_	10,000 yuan
	Space form	Compactness of construction land *X*_12_	%
		Intensity of urban development and utilization *X*_13_	%
		Land use efficiency *X*_14_	%
External environment	Policy guidance	Expenditure of public finance *X*_15_	10,000 yuan
		Expenditure on scientific undertakings *X*_16_	10,000 yuan
	Scale and location	Urban size *X*_17_	/
		Distance to the nearest city above the provincial capital *X*_18_	m

**Table 6:** Comparison of indicators of three models.

Model	RSS	AICc	R^2^	Adjusted R^2^
GR	21.609	112.267	0.926	0.921
GWR	15.340	−42.198	0.952	0.942
MGWR	7.080	−75.343	0.976	0.968

## Data Availability

The data and statistical analysis methods are available upon request from the corresponding author.
